# Extending smoking abstinence after release from smoke-free prisons: protocol for a randomised controlled trial

**DOI:** 10.1186/s40352-016-0046-6

**Published:** 2017-01-23

**Authors:** Cheneal Puljević, Stuart A. Kinner, Dominique de Andrade

**Affiliations:** 1 Griffith Criminology Institute, Griffith University, Brisbane, Australia; 2 Menzies Health Institute Queensland, Griffith University, Brisbane, Australia; 3 Melbourne School of Population and Global Health, University of Melbourne, Melbourne, Australia; 4 Mater Research Institute, University of Queensland, Brisbane, Australia; 5 School of Public Health and Preventive Medicine, Monash University, Melbourne, Australia; 6 Centre for Adolescent Health, Murdoch Childrens Research Institute, Melbourne, Australia; 7 Centre for Youth Substance Abuse Research, Institute of Health and Biomedical Innovation, Centre for Children’s Health Research, Queensland University of Technology, Brisbane, Australia

**Keywords:** Smoking cessation, Tobacco, Forced abstinence, Incarceration, Re-entry, Randomized controlled trial

## Abstract

**Background:**

A smoking ban was implemented across all prisons in Queensland, Australia, in May 2014, with the aim of improving the health of prisoners and prison staff. However, relapse to smoking after release from prison is common. Only one previous study, conducted in the United States, has used a randomised design to evaluate an intervention to assist individuals in remaining abstinent from smoking following release from a smoke-free prison.

**Methods:**

This paper describes the rationale for and design of a randomised controlled trial of an intervention to extend smoking abstinence in men after release from smoke-free prisons in the state of Queensland, Australia. Participants in the intervention group will receive a brief intervention involving four group sessions of motivational interviewing and cognitive behavioural therapy, initiated 4 weeks prior to release from prison. The comparison group will receive a pamphlet and brief verbal intervention at the time of baseline assessment. Assessment of self-reported, post-release smoking status will be conducted by parole officers at regular parole meetings with the primary outcome measured at 1 month post release.

**Discussion:**

The prevalence of smoking and related health harms among people who experience incarceration is extremely high. Effective interventions that result in long-term smoking cessation are needed to reduce existing health disparities in this vulnerable population.

**Trial registration:**

Current Controlled Trials ACTRN12616000314426

## Background

Tobacco smoking is a global public health issue, killing approximately six million people annually (World Health Organisation, 2015). It is a major risk factor for many physical disorders such as coronary heart disease, cancer, and strokes (AIHW, 2014a). In Australia, tobacco is responsible for 7.8% of the total burden of disease and injury—making it the greatest single contributor to the burden of disease in the country (Begg et al., 2007). Fortunately, the health benefits of quitting tobacco smoking are both substantial and rapid (Zwar et al. 2014).

Despite declining levels of tobacco smoking in Australia’s general population (AIHW, 2014b), the prevalence of smoking has remained stubbornly high for Australian prisoners—74% of whom smoke (AIHW, 2015), a rate which is five times that in the general population (AIHW, 2014a). One reason for the high prevalence of smoking in prisoners is that groups with a high prevalence of smoking in the community—such as Indigenous people, people with a mental illness, people who inject drugs, and people from socio-economically disadvantaged backgrounds—are also at increased risk of incarceration (AIHW, 2013a, 2013b, 2015; Belcher et al. 2006). The high prevalence of tobacco consumption among prisoners contributes significantly to increased age-adjusted mortality rates and years of potential life lost, when compared to the general population (Binswanger et al., 2014; Kinner, Forsyth, et al. 2013; Kinner, Lennox, et al. 2013), and to some of the worst health outcomes out of any identifiable population group (Maruschak & Beck, 2001; Richmond et al., 2013a).

In an effort to improve prisoners’ health, smoking bans have been implemented in many prisons around the world. However, despite short-term benefits associated with smoking bans (Binswanger et al., 2014), the majority of prisoners return to smoking upon release (Clarke et al., 2013; Lincoln et al. 2009), suggesting that these bans result in short-term tobacco abstinence only (Donahue, 2009). Furthermore, recent studies (Cropsey & Kristeller, 2005; Kauffman et al. 2011) suggest that not all prisoners adhere to smoking bans. This suggests the need for efforts to promote smoking cessation both in and, critically, after release from prison, to reduce rates of tobacco-related morbidity and mortality among ex-prisoners (Belcher et al., 2006; Djachenko et al. 2015; Gautam et al. 2011; Kauffman et al., 2011; Mackay, 2014).

There is good evidence for the effectiveness of Cognitive Behavioural Therapy (CBT; Killen et al., 2008; Stead & Lancaster, 2005; Webb Hooper et al. 2010) and Motivational Interviewing (MI; Lindson-Hawley et al. 2015) for promoting smoking cessation, particularly when delivered in a group format (Stead & Lancaster, 2005). However, no studies have evaluated the effectiveness of a group-based combined CBT and MI intervention for promoting smoking abstinence in ex-prisoners, and only one previous study has rigorously evaluated an intervention designed to maintain smoking abstinence after release from a smoke-free prison. The Working Inside for Smoking Elimination (WISE) study (Clarke et al., 2013) used a randomised design to evaluate the effectiveness of individual intervention sessions, based on principles of both CBT and MI, for improving smoking abstinence after release from a smoke-free prison in the state of Rhode Island, USA. This study found a modest yet significant effect; 12% of the intervention group remained abstinent from tobacco at 3 months post-release, compared with only 2% of the control group.

The aim of this study is to evaluate the effectiveness of a group-based version of the WISE intervention for maintaining smoking abstinence among men in the first 3 months after release from smoke-free prisons in Queensland, Australia.

## Methods

### Study Design

**Fig. 1:**
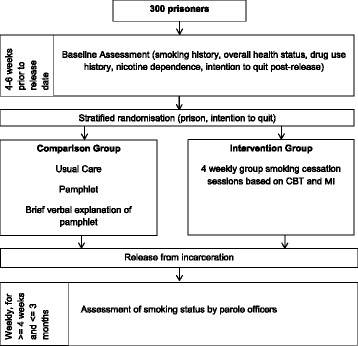
Overview of study design

The study is a randomised controlled trial comparing two groups of soon-to-be released male prisoners. Three hundred sentenced prisoners will be recruited from four correctional centres in Queensland. Intervention group participants will take part in four group sessions of CBT and MI encouraging smoking abstinence post release, in the 4 to 6 weeks prior to release from prison. The comparison group will receive usual care plus a pamphlet encouraging smoking abstinence and a brief verbal explanation of the pamphlet at baseline. After individual baseline screening interviews assessing smoking history and future smoking intentions, participants will be randomised 1:1 to the intervention or comparison group. Once participants are released from custody, parole officers will record their self-reported smoking status at every appointment, for a maximum of 3 months. The primary outcome is point-prevalence of tobacco smoking abstinence at 1 month post-release. This study has been approved by the Griffith University Human Research Ethics Committee and by the Queensland Corrective Services (QCS) Research Committee. The trial is registered with the Australian New Zealand Clinical Trials Registry (ACTRN12616000314426) (Fig. 1).

### Recruitment and inclusion criteria

Three hundred male prisoners will be recruited from four correctional centres in the populous south-east corner of Queensland, Australia. Queensland was the second Australian jurisdiction to implement a smoking ban across all correctional institutions, on 5 May 2014 (Mackay, 2014). Smoking cessation support was provided to staff and prisoners in the form of free nicotine replacement therapy (NRT) and access to telephone counselling.

QCS will identify all prisoners at the four selected correctional centres who fulfil the following eligibility criteria: age 18 years old or older; male; incarcerated after the smoking ban was introduced (5 May 2014); have been incarcerated continuously for at least 4 weeks (so as to have passed tobacco withdrawal); due to be released from custody within 4 to 6 weeks; and will be released to a minimum of 1 month of court-ordered parole.

Eligible prisoners will be identified by prison staff and provided with an information sheet that provides details of the study. If the eligible prisoner is interested in participating, he will be shown to a private interview room, where the researcher will obtain informed consent and undertake the baseline assessment. Randomisation will occur at conclusion of the baseline assessment.

### Groups and intervention

Intervention group participants will receive four weekly group sessions of the intervention in the 4 weeks prior to their release. They will be assigned to a group depending on their release date, ensuring that all members of each group are due for release at a similar time. Each group will consist of between four and six participants. Each intervention session will be approximately 40–60 min in duration, and will be arranged at dates and times that are convenient to the prisoners and prison staff.

Consistent with the WISE study (Clarke et al., 2013), sessions one and four will be based on principles of MI, and sessions two and three on CBT. MI sessions will focus on aiding the development of self-efficacy and personal choice, and on addressing group members’ ambivalence towards remaining abstinent from smoking post-release. The CBT sessions will teach participants to recognise specific environmental and event triggers to smoking and identify behavioural and coping strategies in response to these triggers. The intervention session plan is based on the successful WISE intervention delivered by Clarke et al. (2013) and has been adapted based on recommendations by a clinical psychologist with extensive experience in Australian correctional settings. Sessions will be delivered by the primary researcher, who has a background in psychology and counselling and has received further training in CBT and MI techniques.

Prisoners in the comparison group will receive a pamphlet at the end of the baseline assessment, as well as a brief (1 to 2 min) verbal explanation of the pamphlet. The pamphlet highlights the advantages of staying abstinent from smoking post-release, and provides tips for doing so.

### Sample size calculation

Sample size calculations were informed by the work of Clarke et al. (2013) who, based on a comprehensive review of the literature, conservatively estimated abstinence rates of 23% for the intervention group and 14% for the control group. With alpha of .05 one-tailed and a power level of at least .80, these estimates result in a desired sample size of 292, which we have rounded up to 300 participants. These estimates are considered conservative for two reasons. First, whereas participants in the study by Clarke et al. (2013) did not receive any cessation support from prison authorities, prisoners in Queensland receive a week’s supply of free nicotine patches upon incarceration, associated with long-term smoking abstinence (Stead et al., 2012; Wu et al. 2006). Second, the observed intervention effect in the study by Clarke et al. (2013) was larger than expected (25% vs. 7% at 3 weeks), and the intervention in this trial is an enhanced version of the WISE intervention.

### Randomisation

After baseline assessment, each participant will be randomised 1:1 to the intervention or comparison group. Randomisation will be stratified by prison. The number of participants in each group per prison will reflect the sample distribution across the four prisons. Randomisation will also be stratified by intention to quit, given evidence (Thibodeau et al. 2010) that prisoners who have an intention to quit are more likely to remain abstinent post-release. The randomisation list will be divided into randomly permuted blocks of size 4, 6, or 8. Within each block, an equal number of intervention and comparison conditions will be assigned. Use of random permuted blocks ensures balance during assignment and helps to prevent participants from guessing which condition they have been assigned to (Beller et al. 2002). At this stage, the number of prisoners who intend to remain abstinent from smoking after release is unknown.

### Measures

The baseline assessment will be administered individually to all participants and will take 15–20 min to complete. It measures participants’ smoking history, overall health status, drug use history, nicotine dependence (using the Fagerström Test for Nicotine Dependence; Fagerstrom & Schneider, 1989), and post-release smoking intentions and desires. The assessment was designed by the authors and informed by previous research (e.g. Clarke et al., 2011, 2013; Richmond et al. 2013a, 2013b).

Once participants have been released from prison, parole officers will assess participants’ self-reported smoking status at each appointment, using four questions: 1) Have you smoked since release? 2) If yes, how many weeks ago did you first smoke? 3) Are you currently smoking? 4) If yes, how many cigarettes do you smoke on average per day? In this way, absolute smoking status, duration of smoking abstinence, and intensity of the relapse (e.g., quantity of cigarettes smoked per day) can be measured. These questions also account for situations where a participant may have had a ‘lapse rather than a relapse’ (e.g., temporarily resumed smoking, then returned to abstinence).

Most parolees meet with their parole officers weekly, but some meet fortnightly or monthly. Parole officers will assess parolees’ smoking status at every meeting, but all participants will be assessed at 1 month post-release (the primary endpoint). Parole officers will continue to ask these four questions at each meeting with the participant for up to 3 months post-release. The rationale for a 3-month end point is that the majority of smoking relapses occur within the first 3 months post-release (Ockene et al., 2000). Clarke et al. (2013) also support this relatively brief follow-up period due to the high relapse rates immediately after release. Although restricting the study to parolees may have implications for generalisability, this is offset by an expected low rate of attrition, since most parolees are expected to attend most of their parole meetings.

### Outcomes

The primary outcome of this study is point-prevalence tobacco abstinence at 1 month post-release, measured by self-report. Secondary outcomes include number of weeks to first cigarette after release, amount of tobacco consumed in the event of relapse, and point-prevalence of tobacco abstinence at 3 months post-release. Participants lost to follow-up will be considered non-abstinent—the most conservative assumption.

### Planned Analyses

For the primary outcome, the primary analysis will be an intention to treat analysis, with a *p*-value of <0.05 from a one-tailed chi square test of proportions considered statistically significant. The secondary analysis will be a per protocol analysis, excluding intervention group participants who did not attend at least three of the four intervention sessions. Secondary outcomes will be examined using chi square tests (for categorical variables), independent samples t-tests (for continuous variables) and discrete time survival analysis (for time to relapse).

## Discussion

Correctional smoking bans have been implemented in a number of countries, with the aim of improving the health of both prison staff and prisoners. Prisoners are highly marginalised (AIHW, 2015), smoke tobacco at a much higher rate than the general community (AIHW, 2015), and experience high rates of morbidity and mortality after release from custody (Borschmann et al., 2016; Cutcher et al. 2014; Kinner, Forsyth, et al. 2013; Kinner & Wang, 2014; Thomas et al., 2016; Winter et al., 2015). Tobacco smoking contributes to these poor health outcomes.

However, correctional smoking bans have not stopped people who experience incarceration from smoking—both because a subset are non-adherent while in prison (Cropsey & Kristeller, 2005) and due to very high rates of resumption soon after release from custody (Lincoln et al., 2009). Studies measuring return to tobacco smoking after release from prison in the United States have found that the majority return to smoking on the day of release (Clarke et al., 2013; Lincoln et al., 2009). Ultimately, while correctional smoking bans may reduce prisoners’ smoking in the short term, the evidence suggests that they fail to produce long-term smoking abstinence (Donahue, 2009). The WISE study (Clarke et al., 2013) provides evidence for the effectiveness of an intervention using a combination of CBT and MI for promoting smoking cessation among former prisoners.

The current study aims to investigate the effectiveness of a similar intervention in a different setting: four prisons in Queensland, Australia. There are four other noteworthy differences between this study and Clarke et al. (2013). In the WISE intervention, participants received six sessions of the intervention session; the sessions were delivered individually; comparison group participants watched health-related films; and participants had never been offered any form of NRT while incarcerated. In the proposed study, participants will receive four weekly group intervention sessions as opposed to six, which we believe will be equally effective and may reduce participant dropout; the intervention will be delivered in a group format, which is not only more efficient (and thus more likely to be supported by prison officials), but also an enhancement given evidence that group-based interventions are more effective for smoking cessation when compared to individual sessions (Stead & Lancaster, 2005); comparison group participants will receive a pamphlet and brief verbal intervention focused on smoking cessation, instead of watching a more generic health-related film; and finally, unlike their American counterparts, Queensland prisoners are offered 1 week of free NRT upon incarceration. As NRT use is linked to improved long-term smoking cessation (Wu et al., 2006), and evidence shows equal effectiveness for short- and long-term NRT treatment (Stead et al., 2012), we anticipate higher rates of tobacco smoking abstinence post-release compared to the WISE study.

Although this will be, to the best of our knowledge, the second ever randomised trial of an intervention to promote smoking cessation after release from prison, the protocol has some limitations. First, the trial is unblinded, but given the nature of the intervention and the follow-up, blinding is not feasible in this setting. Second, we note that some degree of contamination between the intervention and comparison groups in the prison setting is likely, but is unfortunately unavoidable in this situation. The consequence of any contamination would be to attenuate the intervention effect, such that the trial would be a conservative test of the intervention. Third, given high rates of reincarceration among ex-prisoners in Australia (Steering Committee for the Review of Government Service Provision, 2016), some proportion of participants may return to prison during follow-up. By making our primary endpoint only 1 month post-release we aim to minimise this form of potentially biased attrition. Fourth, the results may not be generalisable to all ex-prisoners, including to important subgroups such as women, people with an intellectual disability, ex-prisoners not on parole, and remandees (pre-trial detainees). Fifth, we rely on parole officers to collect follow-up data, which is likely to substantially reduce loss to follow-up but, despite the fact that tobacco smoking is not a prohibited behaviour for parolees, may impact on the data quality where good rapport cannot be established between parole officers and parolee participants. Sixth, our outcome will be measured by self-report, without biological verification. Although there is a possibility of self-report bias, tobacco smoking is not prohibited for parolees, and studies using both self-report and tobacco abstinence validation tests (e.g. presence of cotinine in urine) show that self-report can be a reliable measure of smoking abstinence (Clarke et al., 2013; Richmond et al., 2013b; Short et al., 2009).

## Conclusions

This will be the first ever randomised controlled trial of an intervention aiming to promote tobacco smoking abstinence after release from prison in Australia, and the first to trial such an intervention among prisoners who received free NRT upon incarceration. A recent global systematic review identified only 95 RCTs investigating interventions aiming to improve the health of people during imprisonment or in the year after release from prison, and only 32 of these studies examined post-release health outcomes (Kouyoumdjian et al., 2015). This study will therefore provide a valuable contribution to the literature in this area.

Although correctional smoking bans aim to reduce tobacco use in prisoners—a group who suffer disproportionately from tobacco-related harms—the evidence suggests that these bans result in short-term tobacco abstinence only. An intervention such as the one proposed here could provide a unique opportunity to reduce existing health disparities in this vulnerable population.
